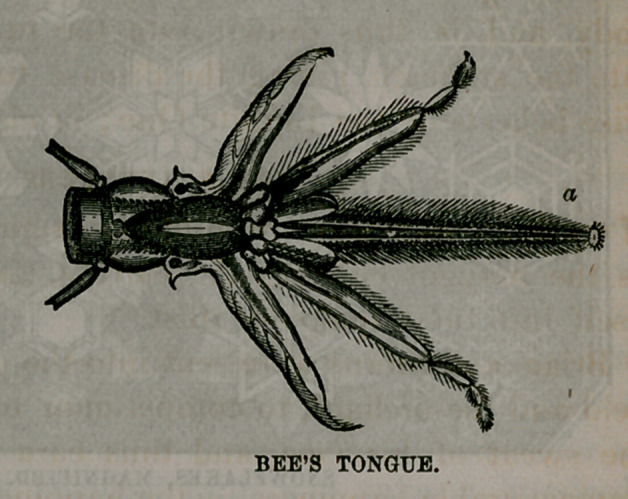# The Microscope

**Published:** 1872-07

**Authors:** 


### THE MICROSCOPE,

Discovered by Janssen, in 1619, meaning the "looking at small things," is a contrivance which enlarges an object to many times its natural size, thus enabling the eye to see what otherwise could not be noticed. One of these useful little instruments, magnifying to the extent of a thousand diameters, that is, making a thing look a thousand times larger than it really is, may be obtained for two or three dollars, and be a source of amusement, instruction, and wonder to a whole family gathered around the fire of a winter's night, affording a varied and most lively entertainment to a whole household, especially the little ones, filling the mind with wonder and admiration of the wisdom and power of Him who " hath done all things well."

The family which makes the fireside attractive is the family in which sons and daughters grow up loving and loved, saved from the contaminations of the street, and from the corrupting influences of bad associations after nightfall; for that is the time when the young are most readily tempted to go astray, as if the darkness covered their wrong doings from human view.

The evening is the best time to use a microscope, as with a lamp, or gas, or candle, the light can be better directed to the exact spot where it is wanted. It is a delightful thing, before a winter's fire, to have the little ones gather around, with inquiring looks and wondering eyes and brightening countenances, so full of joyous expectancy; and then how easy to lead the mind upward, in loving admiration of Him who made all things, from an insect's eye, or a snowflake, to the sun in its glory!

ANT'S EYE, MAGNIFIED.

This cut shows us the eye of an ant. It does not move around, or from side to side, nor even turn its little head, to advise it of coming danger. The eye is firmly fixed, and no more moves than the nose or ears; but in each eye there are multitudes of eye-balls, which face every possible direction, and every image which comes within their scope is perfectly portrayed and seen, whether it be a particle of dust brushed from a feather, or a man, or a mountain.

By this magical instrument, when of great power, insects of various sizes may be seen in the hollow of a grain of sand. Every hair of the head is found to be hollow, and covered with scales, like those of a fish; indeed, these can be felt by drawing one between the finger and a thumb-nail the wrong way, for, in one direction, with the scales, the hair is smooth. Our very bodies are covered with scales, a hundred of which can be covered by a single grain of sand from the sea. Look at a butterfly's wing and its beautiful hues. Every bright spot is made of uncounted little feathers, while the mould on a crust of bread looks like a luxuriant frost. The smallest mite will take a hundred steps in a second. Every leaf is fed upon by millions of miniature cattle, while a drop of stagnant water has uncounted monsters wriggling in it with the freedom of a whale at sea. But it is well to know that running streams and deep wells contain none of these; they are too cold for their life, and the dashing of the waves and the dancing of the waters over pebbly beds are too boisterous for their frail frames.

THE BEAUTIFUL SNOW, so pearly white, so soft and feathery, and to us appearing to be of one universal sameness, when seen under the microscope, as freshly fallen from the sky, is composed of many flakes, yet every flake is different from every other. Of the myriads which fall of a winter's night, no two have yet been found to be alike, except in one amazing point; there is not one that by any chance has a jagged edge, but there is a wonderful evenness and regularity. Every single flake is composed of perfect crystals, and of the most beautiful and varied forms imaginable, as seen in the accompanying cut.

SNOWFLAKES, MAGNIFIED.

In examining the sting of a bee, the point *a* is arrow-shaped, formed like the barb of a fish-hook, easy to enter, but the drawing out tears the flesh and aids in the hurting; at the same instant, through the sting, which is hollow, the poison from the little sacs, *b*, *b*, *c*, is injected, and constitutes the

VENOM OF THE BITE.

On the same principle are formed the bill of the mosquito, and in part the fang of the rattlesnake, which is hollow; and when the sac of poison at the bottom of it is pressed, the poison is forced into the wound which the fang made, there to rankle and rage and destroy.

STING OF A BEE.

In the foot of a fly, the microscope shows a single hair as large as the foot itself, with the marvelous little pads, or soles, which enable it to cling to the ceiling, with its head downwards, all unconscious of its doing anything wonderful.

FLY'S LEG AND FOOT.

BEE'S TONGUE.

The end of the bee's tongue is seen at *a*. It is through this that the substance is drawn up from the flowers which is made into honey and wax, the other parts seeming to be used in steadying the little worker's body while exploring the flowers.

The uses to be made of the microscope in the detection of disease, or in the investigation of diseased conditions of the system, have not yet been determined, as microscopy is still in its infancy. Suffice it to say, that insects, cells, and all forms of vegetable and animal life, are found everywhere, in the body and out of it; in the corners of the eye, in the ear, the nose, in fact, almost everywhere in the

HOUSE WE LIVE IN, a worm for almost every mechanism of the system, making it a pasture-field on which to graze, to flourish, and to thrive.

Of late it has become quite a common thing to examine the discharges connected with various forms of disease; and each one, thus far, seems to have abounded in a specific form of life, either vegetable or animal, forms of amazing fecundity, paralleled only in the process of fermentation, as in the making of a loaf of bread. One kind of life cell has been found in the discharges of

CHOLERA, which begins to rise in the air the instant of leaving the body, and is thus drawn into the lungs, and swallowed into the stomach, giving the disease to others, to work the wise behests of the Infinite One; or else to be

THE SCAVENGERS

of creation, to remove from human sight all that can offend, as the maggot eats up the carrion, then dies, and passes itself into the impalpable dust.

Briars and thorns were sent into the garden and the grain field and the orchard, to compel man to gain his bread by the sweat of his face, and thus earn for himself also an immeasurable happiness, — the happiness of a hearty appetite, a vigorous digestion, delicious sleep, and

GLORIOUS GOOD HEALTH!

We do not yet know but that even the infinitesimal insect which multiplies into a million in a minute, to propagate disease, if it finds dirt to propagate it in, was intended to be the thorn of the animal world, to compel man, the master, to such watchfulness and industries as would banish from his person, his chamber, his dwelling, his garden, his fields, and his pastures, every possible thing which could harbor a disease-producing worm, or which by any possibility could afford filth enough for it to feed upon for a minute.

THE BLUE-BOTTLE FLY

deposits eggs in ulcers and sores, and at the various outlets of the human body, flesh-wounds, etc., and these eggs are developed into worms in a few hours. By cleanliness and care, such a result will be avoided.

COURAGE LUMPS

are reddish pimples on the face of the young, the central point soon becoming yellowish. The above name has been given under the impression that they result from grossness of body, or are connected with the transformation period from youth to manhood. They are often a disfigurement, as well as a source of mortification. Either let them alone, or when ripe, yellow at the top or centre, put a thumb-nail on either side, press inwards and upwards, and a whitish, cheesy, thread-like worm comes away.

MEASLES AND SCARLET FEVER

have associated with their appearance a living germ, as also the common

ITCH.

But whether they cause disease or were intended to prevent it, one point is clear, that it is man's duty, in either case, to prevent the filth which feeds them, or to remove them, with the filth in which they appear, and thus be perfectly clean in his person, in his habitation, and in all his surroundings, that there be no unseemly object anywhere in sight, it being a condition of health of body and purity of mind and heart that the man himself should be pure and clean, as the precedent of his being good, and made fit for angelic associations.

## Figures and Tables

**Figure f1:**
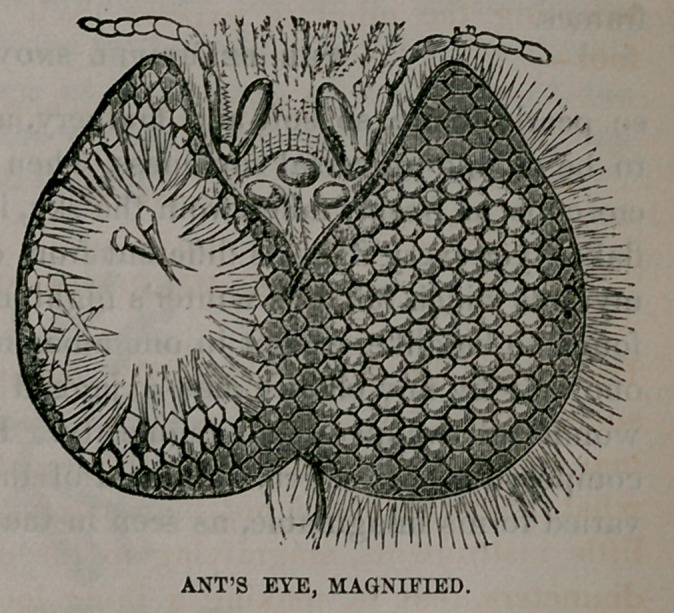


**Figure f2:**
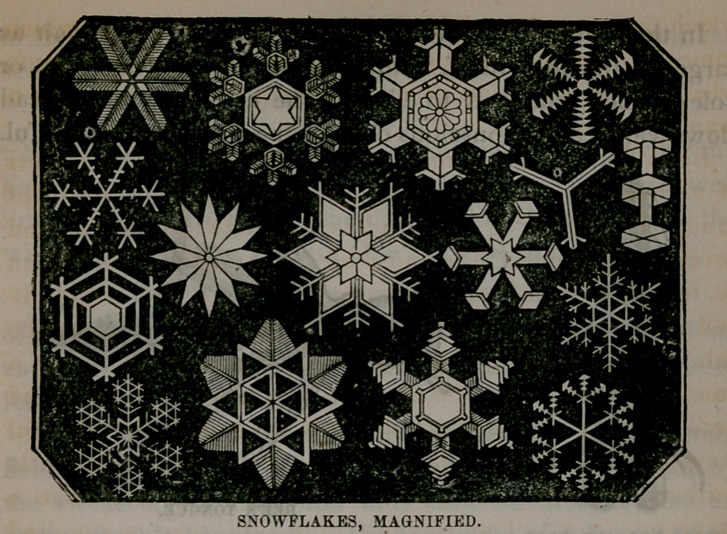


**Figure f3:**
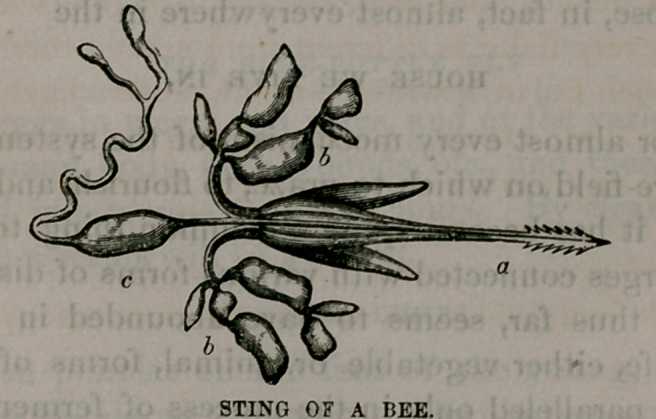


**Figure f4:**
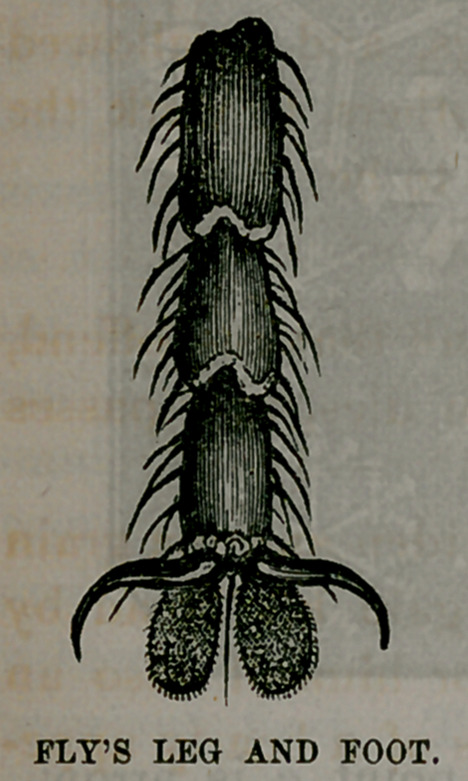


**Figure f5:**